# Novel Dental Cement to Combat Biofilms and Reduce Acids for Orthodontic Applications to Avoid Enamel Demineralization

**DOI:** 10.3390/ma9060413

**Published:** 2016-05-25

**Authors:** Ning Zhang, Mary Anne S. Melo, Joseph M. Antonucci, Nancy J. Lin, Sheng Lin-Gibson, Yuxing Bai, Hockin H.K. Xu

**Affiliations:** 1 Department of Orthodontics, School of Stomatology, Capital Medical University, Beijing 100050, China; dentistzhang112@163.com; 2 Biomaterials & Tissue Engineering Division, Department of Endodontics, Periodontics and Prosthodontics, University of Maryland Dental School, Baltimore, MD 21201, USA; mmelo@umaryland.edu; 3 Biomaterials Group, Biosystems and Biomaterials Division, National Institute of Standards and Technology, Gaithersburg, MD 20899, USA; Joseph.antonucci@nist.gov (J.M.A.); nancy.lin@nist.gov (N.J.L.); sheng.lin-gibson@nist.gov (S.L.-G.); 4 Center for Stem Cell Biology & Regenerative Medicine, University of Maryland School of Medicine, Baltimore, MD 21201, USA; 5 Marlene and Stewart Greenebaum Cancer Center, University of Maryland School of Medicine, Baltimore, MD 21201, USA; 6 Department of Mechanical Engineering, University of Maryland, Baltimore County, MD 21250, USA

**Keywords:** orthodontic cement, protein repellent, antibacterial property, shear bond strength, human saliva microcosm biofilm, white spot lesions

## Abstract

Orthodontic treatments often lead to biofilm buildup and white spot lesions due to enamel demineralization. The objectives of this study were to develop a novel bioactive orthodontic cement to prevent white spot lesions, and to determine the effects of cement compositions on biofilm growth and acid production. 2-methacryloyloxyethyl phosphorylcholine (MPC), nanoparticles of silver (NAg), and dimethylaminohexadecyl methacrylate (DMAHDM) were incorporated into a resin-modified glass ionomer cement (RMGI). Enamel shear bond strength (SBS) was determined. Protein adsorption was determined using a micro bicinchoninic acid method. A dental plaque microcosm biofilm model with human saliva as inoculum was used to investigate metabolic activity, colony-forming units (CFU) and lactic acid production. Incorporating 3% of MPC, 1.5% of DMAHDM, and 0.1% of NAg into RMGI, and immersing in distilled water at 37 °C for 30 days, did not decrease the SBS, compared to control (*p >* 0.1). RMGI with 3% MPC + 1.5% DMAHDM + 0.1% NAg had protein amount that was 1/10 that of control. RMGI with triple agents (MPC + DMAHDM + NAg) had much stronger antibacterial property than using a single agent or double agents (*p* < 0.05). Biofilm CFU on RMGI with triple agents was reduced by more than 3 orders of magnitude, compared to commercial control. Biofilm metabolic activity and acid production were also greatly reduced. In conclusion, adding MPC + DMAHDM + NAg in RMGI substantially inhibited biofilm viability and acid production, without compromising the orthodontic bracket bond strength to enamel. The novel bioactive cement is promising for orthodontic applications to hinder biofilms and plaque buildup and enamel demineralization.

## 1. Introduction

Orthodontic therapies with fixed appliances often lead to biofilm buildup with increased cariogenic bacteria which are difficult to clean around the brackets [1,2]. Biofilm acids can cause enamel demineralization, resulting in white spot lesions or even eventually caries lesions [3]. The incidence of enamel white spot lesions in orthodontic patients was reported to be as high as 50% [4]. Clinical observations showed that the most common sites for white spot lesions are at the junctions of the orthodontic cement and tooth enamel [3]. Orthodontic cements around the brackets tend to accumulate biofilms due to their relatively rough surfaces [2,5]. With increasing demand for esthetics around the world and growing popularity of orthodontic treatments, it would be meritorious to fabricate a new orthodontic cement with bioactive capability to inhibit biofilm buildup and acid production around orthodontic brackets.

Resin-based materials such as composites, cements and adhesives are increasingly used in dentistry [6,7]. Resin-modified glass ionomer cements (RMGIs) have been applied as orthodontic cements because of fluoride (F) ion release and good adhesive strength to enamel [8]. However, previous studies indicated that RMGIs had biofilm buildup around orthodontic brackets due to their rough surfaces and high surface-free energy and polarity [9,10,11]. Antimicrobial agent incorporation in RMGIs could supress bacteria growth on the cement [12]. Recently, poly(quaternary ammonium salt) was incorporated into RMGIs to obtain potent bactericidal functions [13]. The bactericidal efficacy of quaternary ammonium compounds was improved with greater alkyl chain lengths (CL) of the ammonium groups [14]. Recent studies developed several new quaternary ammonium methacrylates (QAMs) having CL ranging from 3 to 18, and these QAMs were added into dental composites and adhesives [15,16]. Among these QAMs, a dimethylaminohexadecyl methacrylate (DMAHDM) with CL of 16 showed the highest bactericidal efficacy. However, there has been no previous report on the incorporation of DMAHDM into RMGIs.

Another bactericidal agent, silver (Ag), has been shown to be effective in supressing a wide range of microorganisms [17,18]. Ag is biocompatible, has low toxicity durable bactericidal properties, and is related to lower bacterial resistance than antibiotics [18,19,20]. In recent studies, nanoparticles of silver (NAg) were developed and added into dental composites [21], bonding agents [22,23] and orthodontic cements [24,25], producing strong bactericidal properties. The present study aimed to combine DMAHDM and NAg together in RMGI for the first time to further enhance the bactericidal potency.

Another approach to supress biofilm growth is to repel protein coverage on the material’s surface. This is because salivary protein coverage on resin surface is a prerequisite for oral bacterial attachment, which relies on salivary proteins on resin to provide anchor points [26,27]. Therefore, developing a protein-repellent orthodontic cement would reduce or eliminate the anchor points for bacteria attachment. Previous studies showed that 2-methacryloyloxyethyl phosphorylcholine (MPC) repelled protein coverage and bacterial attachment [28,29]. Several medical devices containing MPC polymer are available clinically, indicating that MPC is biocompatible [30,31]. Recently, MPC was incorporated into dental composite, adhesive and RMGI, which were able to repel proteins [32,33,34]. Incorporation of protein-repellent MPC and antibacterial NAg together into an orthodontic cement greatly reduced biofilm growth, compared to that using MPC or NAg alone [34]. However, a search of the literature to date showed no report on combining triple agents (MPC, DMAHDM, and NAg) into orthodontic cement to maximize the biofilm-inhibition capability.

The aims of this study were to synthesize a bioactive orthodontic cement to inhibit enamel white spot lesions and investigate the effects of adding MPC, DMAHDM and NAg in RMGI on enamel bond strength, human saliva microcosm biofilm growth and lactic acid production. It was hypothesized that: (1) RMGI containing MPC, DMAHDM and NAg individually or in combination would not affect the enamel bond strength, compared to RMGI control; (2) the new cement would adsorb much less protein than control; (3) the cement using triple agents of MPC, DMAHDM and NAg would have much stronger anti-biofilm property than using single agent or double agents.

## 2. Materials and Methods

### 2.1. MPC Incorporation into RMGI

A RMGI (Vitremer, 3M, St. Paul, MN, USA), referred to as VT, was used as the parent system. VT contained fluorosilicate glass particles and a photo-curable polyalkenoic acid liguid. VT is recommended for use in Class III, V and root-caries restorations, Class I and II in primary teeth, core-buildup, and as an orthodontic cement [9,10]. The manufacturer suggested a powder:liquid mass ratio of 2.5:1. VT was selected because RMGIs are used as orthodontic cements due to their F-release and good adhesion strength to enamel [9,10]. The purpose was to test a model system here; the triple agent incorporation method can then be applied to other orthodontic cements.

MPC (Sigma-Aldrich, St. Louis, MO, USA) was incorporated into VT at MPC/(VT + MPC) mass fraction of 3%. A previous study indicated that 3% MPC produced a potent protein-repellent activity without decreasing the bond strength [34].

### 2.2. NAg Incorporation into RMGI

0.1 g of Silver 2-ethylhexanoate (Strem, Newburyport, MA, USA) was dissolved into 0.9 g of 2-(tert-butylamino)ethyl meth-acrylate (TBAEMA, Sigma-Aldrich, St. Louis, MO, USA) [21,22]. TBAEMA enhanced the solubility by creating Ag-N bonds with Ag ions to aid Ag salt to dissolve in the resin solution [21,22]. TBAEMA has reactive methacrylate groups which can be bonded chemically in the resin matrix when it is light-cured. The Ag solution was added into VT at a silver 2-ethylhexanoate/(VT + silver 2-ethylhexanoate) mass fraction of 0.1%. This ratio was shown to achieve a strong bactericidal effect, without decreasing the mechanical strength of the resin, based on previous studies [21,22].

### 2.3. DMAHDM Incorporation into RMGI

The synthesis of DMAHDM was based on a modified Menschutkin reaction in which a tertiary amine was reacted with an organo-halide [15]. Briefly, of 2-(dimethylamino) ethyl methacrylate (DMAEMA, Sigma-Aldrich, St. Louis, MO, USA) of 10 mmol and 10 mmol of 1-bromohexadecane (BHD, TCI America, Portland, OR, USA) were combined with 3 g of ethanol in a 20 mL scintillation vial, which was stirred at 70 °C for 24 h for the reaction to complete. Then, the solvent was evaporated, and DMAHDM was obtained as a clear, colorless, and viscous liquid [15]. DMAHDM was added into VT at a DMAHDM/(VT + DMAHDM) mass fraction of 1.5%. DMAHDM mass fractions ≥2% were not used, due to the enamel bond strength was decreased when 2% DMAHDM was combined with 3% MPC and 0.1% NAg in preliminary study.

Another orthodontic adhesive (Transbond XT, 3M, Monrovia, CA, USA) served as a control (referred to as TB control). TB contained silane-treated quartz particles at 70%–80% by mass, 10%–20% bisphenol-A-diglycidyl ether dimethacrylate, 5%–10% bisphenol-A-bis (2-hydroxyethyl) dimethacrylate, <2% silane-treated silica, and <0.2% diphenyliodonium hexafluorophosphate. TB had higher enamel bond strength [35,36]. VT had acceptable enamel bond strength for orthodontic brackets [9,10]. MPC, DMAHDM and NAg were incorporated into VT, but not into TB, because TB had no fluoride release. The purpose here was to formulate a fluoride-releasing orthodontic cement possessing bactericidal and protein-repellent functions.

Hence, the following nine orthodontic cements were investigated:

(1) **Non-bioactive control:** Transbond XT control (referred to as TB control);
(2) **F-releasing control:** Vitremer control (referred to as VT control);
(3) **Protein-repellent group:** 97% Vitremer + 3% MPC (referred to as VT + MPC);
(4) **QAM group:** 98.5% Vitremer + 1.5% DMAHDM (referred to as VT + DMAHDM);
(5) **Nano-silver group:** 99.9% Vitremer + 0.1% NAg (referred to as VT + NAg);
(6) **Protein-repellent + QAM group:** 95.5% Vitremer + 3% MPC + 1.5% DMAHDM (referred to as VT + MPC + DMAHDM);
(7) **Protein-repellent + Nano-silver group:** 96.9% Vitremer + 3% MPC + 0.1% NAg (referred to as VT + MPC + NAg);
(8) **QAM + Nano-silver group:** 98.4% Vitremer + 1.5% DMAHDM + 0.1% NAg (referred to as VT + DMAHDM + NAg);
(9) **Protein-repellent + QAM + Nano-silver group:** 95.4% Vitremer + 3% MPC + 1.5% DMAHDM + 0.1% NAg (referred to as VT + MPC + DMAHDM + NAg).

### 2.4. Enamel Shear Bond Strength (SBS) and Adhesive Remnant Index (ARI)

According to the above nine orthodontic cements, 180 extracted human first premolars with intact buccal surface were randomly divided into 9 groups of 20 teeth in each group. The tooth was vertically placed in acrylic resin (Lang Dental, Wheeling, IL, USA) so that the buccal surface of the tooth could be parallel to the applied force when the subsequent shear bond test. Commercial orthodontic brackets (Ormco Series 2000, Sybron Dental, Orange, CA, USA) were used.

For group 1, according to the manufacturers’ instructions, 37% phosphoric acid (Scotchbond, 3M ESPE, St. Paul, MN, USA) was used to etch enamel for 30 s and then rinsed for 10 s. Enamel was gently dried with a stream of air until it became whitish [37]. The etched enamel was coated with primer of Transbond XT in a thin and uniform coating. Then, the adhesive paste of Transbond XT was applied to bracket base and pushed against the etched enamel. A force of 3 N was vertically placed on the bracket for 5 s using a force gauge (Correx, Bern, Switzerland) [38]. A clinical probe was used to remove excess adhesive around the bracket base The specimen was light-polymerized (Demetron VCL 401, Demetron, CA, USA) for a total of 40 s, on the mesial aspect for 20 s and on the distal aspect for 20 s [38,39].

For groups 2–9, based on the literature research, RMGI could serve as orthodontic cement without acid etching the enamel [37,38,39]. Each tooth was cleaned and wiped with a moist cotton roll to ensure that the enamel surface was neither desiccated nor having too much water [37,39]. A VT paste was used to the bracket base. The bracket was bonded to the enamel. The bracket was light-polymerized for a total of 40 s as described above. Each group was randomly divided into two subgroups of 10 specimens each, and immersed in distilled water at 37 °C for 1 day or 30 days, respectively.

For shear bond strength testing, a chisel was connected to a Universal Testing Machine (MTS, Eden Prairie, MN, USA) and the chisel tip was placed on the top part of the bracket. A force was applied to the bracket at a displacement rate of 0.5 mm/min until the bond failed [37,39]. Enamel shear bond strength SBS was calculated as the force at bond failure divided by the bracket surface area [37,38,39].

After debonding, each enamel surface was examined with a stereomicroscope (Zoom 2000, Leica, Wetzlar, Germany). The ARI scores were determined as defined previously [40]. These scores are correlated to the remnant resin amounts on enamel to indicate where fracture had occurred during the bond testing [37,39]. The following scores were used. 0 = no cement remained on enamel. 1 = less than half of the cement remained on enamel. 2 = more than half of the cement remained on enamel. 3 = all the cement remained on enamel.

### 2.5. Measurement of Protein Adsorption onto Orthodontic Cement Surface

For protein-repellent and anti-biofilm experiments, each cement paste was placed into disk molds of 9 mm in diameter and 2 mm in thickness [15,34], and light-polymerized for 40 s on each open side. The cured disks were stored in 200 mL of water and stirred with a magnetic bar at 100 rpm for 1 h to remove any uncured monomers, following previous studies [41,42]. The samples were then sterilized with ethylene oxide (Anprolene AN 74i, Andersen, Haw River, NC, USA) and de-gassed for 3 days [15,16].

A micro bicinchoninic acid (BCA) method was used to measure protein adsorption on cement disk surface [34,43]. Each disk was stored in phosphate buffered saline (PBS) for 2 h. The disks were stored in bovine serum albumin (BSA) (Sigma-Aldrich, St. Louis, MO, USA) solution at 37 °C for 2 h. The protein solution had BSA at a concentration of 4.5 g/L, following a previous study [43]. The disks then were rinsed with fresh PBS by stirring at 300 rpm for 5 min (Bellco Glass, Vineland, NJ, USA), stored in sodium dodecyl sulfate (SDS) at 1% in PBS, and sonicated for 20 min to detach the BSA from the sample surfaces. The BSA concentration in the SDS solution was measured using a protein analysis kit (micro BCA protein assay, Fisher Scientific, Pittsburgh, PA, USA). The protein adsorption on the sample was calculated via the protein concentration [34,43]. Six samples were used for each group.

### 2.6. Saliva Collection for Biofilm Inoculum

Using human saliva for growing dental plaque microcosm biofilms is meritorious in keeping the complexity and heterogeneity of the dental plaque *in vivo* [44]. Saliva was collected from ten healthy adult donors having natural dentition without active caries and not having used antibiotics within the preceding 3 months [15,16]. The donors did not brush teeth for 24 h and abstained from food and drink intake for 2 h prior to donating saliva [45]. The saliva was added, with 1:50 final dilution, to the McBain artificial saliva medium as inoculum. The medium contained mucin (type II, porcine, gastric) at a concentration of 2.5 g/L; bacteriological peptone, 2.0 g/L; tryptone, 2.0 g/L; yeast extract, 1.0 g/L; NaCl, 0.35 g/L, KCl, 0.2 g/L; CaCl_2_, 0.2 g/L; cysteine hydrochloride, 0.1 g/L; hemin, 0.001 g/L; vitamin K1, 0.0002 g/L, at pH 7 [46].

### 2.7. Live/Dead Staining of Biofilms Grown on Orthodontic Cements

Orthodontic cement disks was placed into a well of 24-well plates, and 1.5 mL inoculum was added. A 2-day incubation formed mature biofilms [32,33,34]. Disks with 2-day biofilms were washed with PBS and stained using the BacLight live/dead kit (Molecular Probes, Eugene, OR, USA) [15,16]. The stained disks were examined using an inverted epifluorescence microscope (Eclipse TE2000-S, Nikon, Melville, NY, USA). Three fields of view were randomly photographed for each disk, which provided a total of 18 images for each orthodontic cement.

### 2.8. MTT Assay of Metabolic Activity of Biofilms on Orthodontic Cements

Cement disks with adherent biofilms grown for 2 days were transferred to a new 24-well plate and 1 mL of MTT [3-(4,5-Dimethylthiazol-2-yl)-2,5-diphenyltetrazolium bromide] was added to each well and incubated for 1 h [15,16]. Then, Disks were transferred to a new 24-well plate and 1 mL of dimethyl sulfoxide (DMSO) was added to solubilize the formazan crystals. The plate was incubated for 20 min and then 200 µL of the DMSO solution from each well was collected, and its absorbance at 540 nm was measured via a microplate reader (SpectraMax M5, Molecular Devices, Sunnyvale, CA, USA) [15,16]. A greater formazan concentration is related to a higher absorbance value, which indicates a greater biofilm metabolic activity growing on the orthodontic cement disk [15,16].

### 2.9. Lactic Acid Production by Biofilms on Orthodontic Cements

Orthodontic cement disks with adherent biofilms grown for 2 days were rinsed with cysteine peptone water (CPW) to remove loose bacteria, and the disks were then placed in a 24-well plate [22,23]. Buffered peptone water (BPW) of 1.5 mL was supplemented with 0.2% sucrose and added to each well. Samples were cultured at 37 °C in 5% CO_2_ for 3 h to allow the bacteria to produce acid. The lactate concentrations in the BPW solutions were measured via an enzymatic lactate dehydrogenase technique following previous studies [22,23]. The absorbance values at 340 nm for the collected BPW solutions were measured using the microplate reader. Standard curves were constructed by employing a lactic acid standard (Supelco, Bellefonte, PA, USA) [22,23].

### 2.10. Colony-Forming Unit (CFU) Counts of Biofilms on Orthodontic Cements

Cement disks with adherent biofilms grown for 2 days were placed in tubes with 2 mL of CPW. The biofilms were collected by using a sonication and vortexing method (Fisher, Pittsburgh, PA, USA) [15,16]. The CFU counts were evaluated by three different kinds of agar plates. First, the total microorganisms was evalated by tryptic soy blood agar culture plates [46]. Second, mitis salivarius agar (MSA) culture plates plusing 15% sucrose were prepared to measure the total streptococci [47]. Third, MSA agar culture plates with the addition of 0.2 units/mL of bacitracin was prepared to measure the mutans streptococci [46].

### 2.11. Statistical Analysis

One-way analyse of variance (ANOVA) was performed to detect the significant effects of the different orthodontic cements on protein-repellent and antibacterial properties. Two-way ANOVA was performed to detect the significant effects of the different orthodontic cements *vs.* water-aging on SBS. Tukey’s multiple comparison test was used to compare the data at a p value of 0.05. The chi-square test was used to evaluate the ARI scores.

## 3. Results

**Figure 1:**
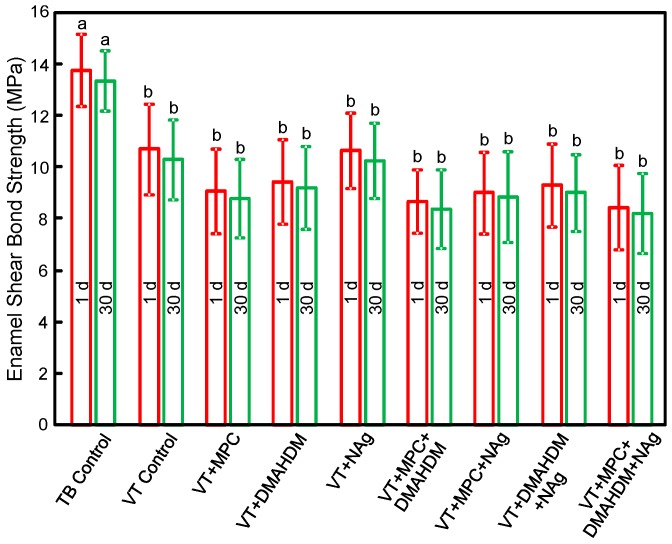
Enamel shear bond strengths (SBS) (mean ± SD; *n* = 10). MPC + DMAHDM + NAg in VT did not adversely affect the SBS, compared to VT control (*p* > 0.1). Water-aging for 30 days had no significant effect on SBS, compared to 1 day (*p* > 0.1). Bars with dissimilar letters indicate values that are significantly different from each other (*p* < 0.05).

The enamel shear bond strengths (SBS) of orthodontic cements are plotted in Figure 1 (mean ± SD; *n* = 10). TB had the greatest SBS. Incorporating 3% MPC, 1.5% DMAHDM and 0.1% NAg into VT did not decrease the SBS, compared to VT control (*p* > 0.1). Water-aging for 30 days caused no significant decrease in SBS, compared to those at 1 day (*p* > 0.1).

The ARI scores from enamel shear bond testing are listed in Table 1 (*n* = 10).TB control had the greatest ARI scores. ARI scores of groups 2–9 were lower than that of TB control (*p* < 0.05). Failures for most TB specimens occurred at the bracket-cement interfaces. The most common site of failure for groups 2–9 was cement-enamel interface. For each material, there was no significant difference between 1 day and 30 days (*p >* 0.1).

**Figure 2:**
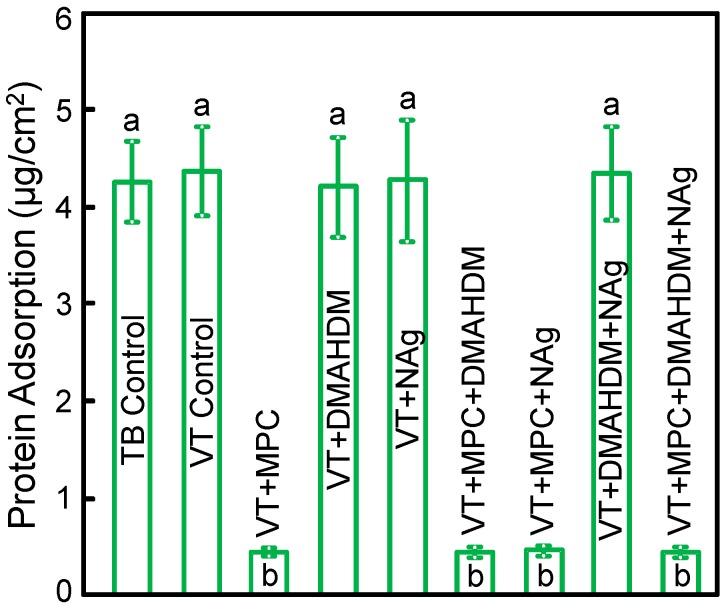
Protein adsorption onto disk surfaces (mean ± SD; *n* = 6).VT + MPC + DMAHDM + NAg had protein adsorption that was an order of magnitude less than that of commercial controls (*p <* 0.05). Bars with dissimilar letters indicate values that are significantly different from each other (*p <* 0.05).

The measured protein amounts on orthodontic cement disks are plotted in Figure 2 (mean ± SD; *n* = 6). Incorporating 3% MPC into VT greatly reduced the protein amount (*p* < 0.05). Adding DMAHDM or NAg into VT did not change protein adsorption. VT + MPC + DMAHDM + NAg had protein amount similar to VT + MPC, which was about 1/10 that of controls (*p* < 0.05).

**Figure 3:**
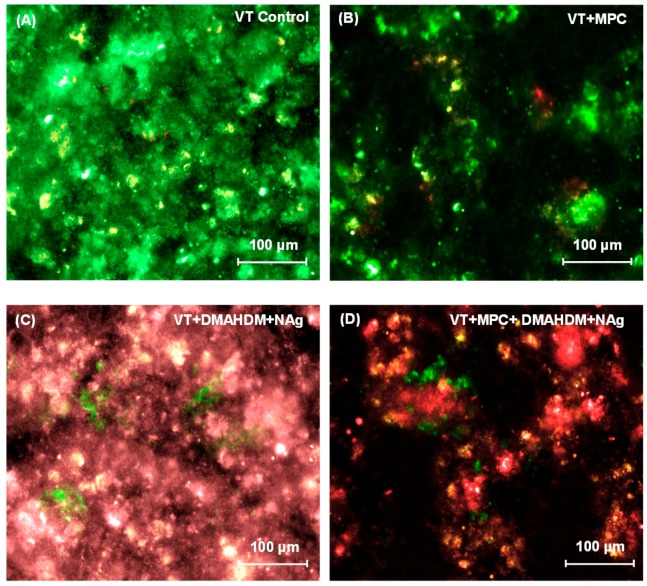
Representative live/dead images of 2-day biofilms on: (**A**) VT control; (**B**) VT + MPC; (**C**) VT + DMAHDM + NAg; and (**D**) VT + MPC + DMAHDM + NAg. Live bacteria were stained green, and compromised bacteria were stained red. When live and dead bacteria were in close proximity or on the top of each other, the staining had yellow or orange colors. VT + MPC + DMAHDM + NAg had less bacterial adhesion, and the biofilms consisted of primarily compromised bacteria.

Representative live/dead images of 2-day biofilms on orthodontic cement disks are shown in Figure 3. In Figure 3A, VT had mostly live bacteria. Biofilms on TB control (not shown) were similar to that in Figure 3A. In Figure 3B, VT + MPC had much less bacterial attachment. In Figure 3C, VT + DMAHDM + NAg had substantial amounts of dead bacteria with red staining. In Figure 3D, VT + MPC + DMAHDM + NAg had less bacterial coverage, and the biofilms consisted of mostly compromised bacteria.

**Figure 4:**
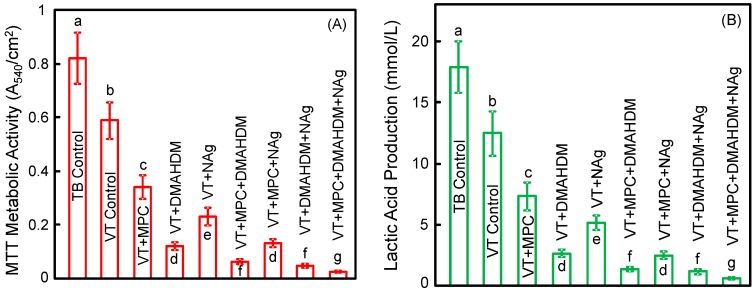
Quantitative viability of 2-day biofilms on disks: (**A**) metabolic activity; (**B**) lactic acid production (mean ± SD; *n* = 6). Biofilms on VT + MPC + DMAHDM + NAg had the least metabolic activity and lactic acid among all groups. In each plot, values with dissimilar letters are significantly different from each other (*p <* 0.05).

Figure 4 plots (A) metabolic activity and (B) lactic acid production of 2-day biofilms on orthodontic cement disks(mean ± SD; *n* = 6). The biofilms on TB control had the greatest metabolic activity and the greatest lactic acid production. Incorporating a single agent of MPC, or DMAHDM, or NAg greatly reduced the lactic acid production and metabolic activity, compared to controls (*p <* 0.05). Incorporating double agents further reduced the lactic acid production and metabolic activity. Triple agents MPC + DMAHDM + NAg in VT had the most potent bactericidal effect. Biofilms on VT + MPC + DMAHDM + NAg had the smallest metabolic activity and produced the least amount of lactic acid (*p <* 0.05) among all groups.

**Figure 5:**
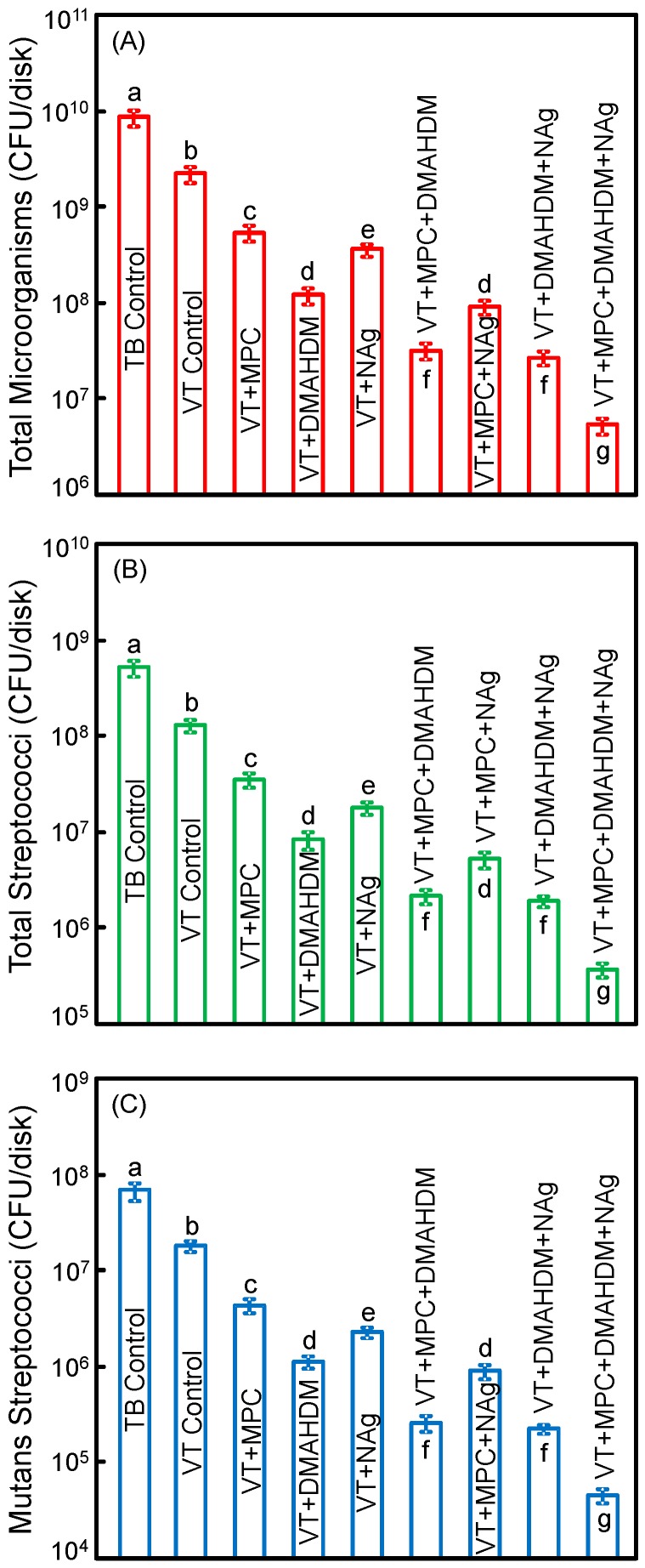
Colony-forming units (CFU) of 2-day biofilms on disks: (**A**) total microorganisms; (**B**) total streptococci; and (**C**) mutans streptococci (mean ± SD; *n* = 6). All three CFU counts on VT + MPC + DMAHDM + NAg were reduced by more than 3 log, compared to TB control. VT + MPC + DMAHDM + NAg had much less CFU than using a single agent.

Figure 5 plots the CFU of biofilms grown for 2 days on orthodontic cement disks for: (A) total microorganisms; (B) total streptococci; and (C) mutans streptococci (mean ± SD; *n* = 6). The values are shown in a log scale. VT control with fluoride release had lower CFU than TB (*p* < 0.05). Adding a single agent of MPC, DMAHDM or NAg decreased the CFU, compared to VT control (*p <* 0.05). Adding double agents further decreased the CFU. VT with triple agents MPC + DMAHDM + NAg had the least CFU among all groups (*p <* 0.05). VT + MPC + DMAHDM + NAg decreased the total microorganisms, total streptococci and mutans streptococci CFU by more than 3 log, compared to TB control.

## 4. Discussion

The present study developed a bioactive orthodontic dental cement via incorporation of triple agents MPC, DMAHDM and NAg in a RMGI for the first time. The rationale for this material design was for the orthodontic cement to have not only F release, but also protein-repellent and antibacterial capabilities to inhibit biofilms and prevent enamel demineralization around orthodontic brackets. VT + MPC + DMAHDM + NAg had strong protein-repellent ability and antibacterial property. Testing the various compositions of cements showed that MPC could repel proteins but had no antibacterial effect; DMAHDM and NAg could kill bacteria but could not repel proteins; and the application of triple agents MPC + DMAHDM + NAg inhibited biofilm viability to a much greater extent than using a single agent or double agents. The benefits were obtained without affecting the enamel bond strength. Therefore, VT + MPC + DMAHDM + NAg is promising for orthodontic applications to combat enamel demineralization and white spot lesions.

Among all the orthodontic cements currently used clinically, RMGIs were found to be the most cariostatic and slightly antibacterial due to their F-release, which could promote remineralization, reduce demineralization and reduce biofilms [9,10]. The present study indeed confirmed that VT had less biofilm CFU than TB. However, clinical surveys found that white spot lesions were still present in fixed orthodontic treatment [3,4], indicating that the F-release from RMGIs did not reduce bacterial growth and acid production sufficiently [48,49]. Therefore, there is a need to modify the RMGI to be able to more strongly inhibit biofilm acids; this was achieved by using a combination of protein-repellent and anti-biofilm antibacterial agents in the present study.

Salivary protein deposit on a material’s surface is the first step for bacterial attachment and biofilm formation [26,27]. It was reported that hydrophilic surfaces are more resistant to protein adsorption than hydrophobic surfaces [21]. MPC polymer was shown to be highly hydrophilic [27]. There is abundant free water but no bound water in the hydrated MPC polymer. The bound water would increase protein adsorption [28,29]. The abundant free water around the phosphorylcholine group could help detach proteins, thereby to repel protein deposit [29,50]. In the present study, VT + MPC adsorbed only 1/10 of the amount of protein of commercial controls. The present study confirmed that VT + MPC indeed had much less bacteria attachment. Therefore, repelling proteins did contribute to a decreased bacterial attachment.

QAMs are promising for dental applications including composites and bonding agents [51,52]. The mode of killing of bacteria via QAMs was related to quaternary ammonium causing bacteria lysis by binding to cell membranes, leading to cytoplasmic leakage [52]. When bacterial cells with negative charges contact a QAM resin with positive charges, the contact could disturb the electric balance of cell membranes thereby leading to membrane breakdown [52]. Long cationic polymers can penetrate bacterial cells to disrupt membranes, like a needle bursting a balloon [53]. DMAHDM with an alkyl chain length of 16 demonstrated a strong bactericidal property [15]. In addition, Ag is an effective bactericidal agent against a wide range of microorganisms [17,18]. The mode of killing was suggested to be Ag ions inactivating the vital enzymes of bacteria to damage the replication ability of bacterial DNA, thereby causing cell death [17,54]. The NAg could release more Ag ions at a low filler level due to their nanoparticle size and great surface area, thus using a low Ag particle filler level for a high efficacy [22,23]. This is desirable for dental applications because low Ag filler levels in the material would not adversely compromise the material mechanical properties and color.

There are two advantages for combining MPC with DMAHDM and NAg in RMGI. First, adding the dual agents DMAHDM and NAg into VT reached stronger antibacterial properties than using DMAHDM or NAg alone. The decrease in biofilms by the two agents appeared to be synergistic and having a multiplying effect. For biofilm metabolic activity, the results indicated that DMAHDM alone in VT reduced the MTT activity to about 1/5 that of VT control. NAg alone in VT reduced the MTT to nearly 1/3 that of VT control. Hence, the multiplying effect would predict that the metabolic activity of VT with both DMAHDM and NAg would reduce the MTT activity to 1/15 of that of VT control. This prediction was confirmed by the present results in Figure 4. This multiplying effect also existed in CFU and lactic acid production. These results suggest that DMAHDM and NAg as double bactericidal agents in RMGI had a multiplying effect in inhibiting biofilm growth.

Second, the mode of antibacterial action of QAMs is contact-inhibition [52]. When a salivary protein pellicle separates the material surface from the overlaying biofilm, the material’s bactericidal property would be decreased [55,56]. It was reported that salivary protein coverage on a NAg-containing material could also lower its bactericidal property [56]. This was likely because the mode of killing of a NAg-containing material involved Ag ion release [17,54]. Salivary protein could capture the positively-charged Ag ions and serve as a barrier to reduce Ag ion release [56]. Because MPC can greatly decrease the protein coverage on the material’s surface, it could thereby improve the bactericidal function of DMAHDM and NAg, and hence provide a synergistic effect on killing the biofilms. Indeed, this synergistic effect was confirmed in the results of Figure 3, Figure 4 and Figure 5, showing that VT + MPC + DMAHDM and VT + MPC + NAg had greater bactericidal potency than those using DMAHDM or NAg without MPC. This synergistic effect agreed with the fact that VT + MPC + DMAHDM + NAg had the least biofilm CFU and the least amount of acid production. In addition, it is important for the orthodontic cement to have a clinically-acceptable bond strength to enamel [35,36,37]. It has been suggested that a metal bracket’s bond strength to enamel should be around 8–9 MPa to possess an adequate bond strength, and yet not too strongly bonded in order to be able to debond at the end of treatment [57]. Enamel is usually acid etched in the process of bonding orthodontic brackets to the teeth [35,36,37]. However, acid-etching the enamel has disadvantages such as multiple steps and leaving resin residue on enamel that cannot be easily removed after debonding [58,59]. RMGIs have the advantage of not needing the acid etch, because the ionic bonding between the hydroxyapatite in enamel and the carboxyl groups of polyalkenoic acid [35,36,37]. Even without acid-etching, groups 2–9 achieved bond strengths that were within the acceptable range. In addition, adding MPC, DMAHDM and NAg into VT did not decrease the SBS, compared to VT control. The ARI data showed that TB control had the greatest ARI scores, while groups 2–9 had ARI scores much less than TB. These findings indicate that the predominant debonding mode of TB control was at the bracket-cement interface, leaving more than half of the cement residual on the enamel surface [40]. In contrast, the bracket debonding of groups 2–9 occurred at the cement-enamel interface. This would be meritorious in the clinic, because it would be easier to clean the tooth with less residual cement for groups 2–9 [35,36,37]. It should be noted that there is very limited clinical data to confirm that the SBS of RMGI is sufficient in the long-term. In fact, an article on a clinical pilot study suggests that bracket retention with RMGI may well be inferior to conventional acid-etched composite resin adhesive [60]. Further studies are needed to evaluate the effect of VT + MPC + DMAHDM + NAg on orthodontic bracket bond strength and its sustainability in a clinical manner, and to test biofilm growth and enamel demineralization under *in vivo* conditions.

## 5. Conclusions

The present study developed a novel bioactive dental cement for orthodontic applications to combat enamel demineralization and white spot lesions. The material design relied on combining MPC to reduce protein coverage, and DMAHDM and NAg for bactericidal properties in RMGI which had fluoride release. The hypotheses were verified that RMGI with MPC + DMAHDM + NAg greatly decreased the protein adsorption and biofilm viability. This was achieved without decreasing enamel shear bond strength. The application of triple bioactive agents was significantly more effective than double agents or single agent in suppressing biofilms and decreasing acids. This novel dental cement is promising for orthodontic applications to inhibit biofilm growth and avert white spot lesions. The method of incorporating triple agents (MPC + DMAHDM + NAg) for synergistic effects may be applicable to a wide range of dental bonding agents, cements, sealants and composites to inhibit biofilm acids and caries.

## Figures and Tables

**Table 1:** ARI Scores of Orthodontic Cements (*n* = 10).

Group	Water-Aging	ARI Scores				Sig. *
		0	1	2	3	
TB control	1 day	0	0	6	4	a
VT control	1 day	4	6	0	0	b
VT + MPC	1 day	4	6	0	0	b
VT + DMAHDM	1 day	3	7	0	0	b
VT + NAg	1 day	4	6	0	0	b
VT + MPC + DMAHDM	1 day	4	6	0	0	b
VT + MPC + NAg	1 day	4	6	0	0	b
VT + DMAHDM + NAg	1 day	3	7	0	0	b
VT + MPC + DMAHDM + NAg	1 day	5	5	0	0	b
TB control	30 days	0	1	8	1	a
VT control	30 days	4	6	0	0	b
VT + MPC	30 days	5	5	0	0	b
VT + DMAHDM	30 days	4	6	0	0	b
VT + NAg	30 days	3	7	0	0	b
VT + MPC + DMAHDM	30 days	4	6	0	0	b
VT + MPC + NAg	30 days	4	6	0	0	b
VT + DMAHDM + NAg	30 days	3	7	0	0	b
VT + MPC + DMAHDM + NAg	30 days	5	5	0	0	b

* Sig. refers to statistical significance, with different letters (a, b) indicating significant differences in ARI scores (*p* < 0.05).

## Disclaimer

Official contribution of the National Institute of Standards and Technology (NIST); not subject to copyright in the United States. Certain commercial materials and equipment are identified in this article to specify the experimental procedure. This does not imply recommendation or endorsement by NIST or that the material or equipment identified is necessarily the best available for the purpose.

## References

[1] Do Nascimento L.E., Pithon M.M., dos Santos R.L., Freitas A.O., Alviano D.S., Nojima L.I., Nojima M.C., Ruellas A.C. (2013). Colonization of Streptococcus mutans on esthetic brackets: Self-ligating *vs.* conventional. Am. J. Orthod. Dentofac. Orthop..

[2] Lim B.S., Lee S.J., Lee J.W., Ahn S.J. (2008). Quantitative analysis of adhesion of cariogenic streptococci to orthodontic raw materials. Am. J. Orthod. Dentofac. Orthop..

[3] Chapman J.A., Roberts W.E., Eckert G.J., Kula K.S., González-Cabezas C. (2010). Risk factors for incidence and severity of white spot lesions during treatment with fixed orthodontic appliances. Am. J. Orthod. Dentofac. Orthop..

[4] Tufekci E., Dixon J.S., Gunsolley J.C., Lindauer S.J. (2011). Prevalence of white spot lesions during orthodontic treatment with fixed appliances. Angle Orthod..

[5] Enaia M., Bock N., Ruf S. (2011). White-spot lesions during multibracket appliance treatment: A challenge for clinical excellence. Am. J. Orthod. Dentofac. Orthop..

[6] Della Bona A., Watts D.C. (2012). Discussing the future of dental materials, processes and products. Dent. Mater..

[7] Della Bona A., Watts D.C. (2013). Evidence-based dentistry and the need for clinically relevant models to predict material performance. Dent. Mater..

[8] Rogers S., Chadwick B., Treasure E. (2010). Fluoride-containing orthodontic adhesives and decalcification in patients with fixed appliances: A systematic review. Am. J. Orthod. Dentofac. Orthop..

[9] Badawi H., Evans R.D., Wilson M., Ready D., Noar J.H., Pratten J. (2003). The effect of orthodontic bonding materials on dental plaque accumulation and composition *in vitro*. Biomaterials.

[10] Ahn S.J., Lim B.S., Lee S.J. (2010). Surface characteristics of orthodontic adhesives and effects on streptococcal adhesion. Am. J. Orthod. Dentofac. Orthop..

[11] Lynch C.D. (2013). Summary of: A retrospective, practice-based, clinical evaluation of Fuji IX restorations aged over five years placed in load-bearing cavities. Br. Dent. J..

[12] Ferracane J.L., Giannobile W.V. (2014). Novel biomaterials and technologies for the dental, oral, and craniofacial structures. J. Dent. Res..

[13] Xie D., Weng Y., Guo X., Zhao J., Gregory R.L., Zheng C. (2011). Preparation and evaluation of a novel glass-ionomer cement with antibacterial functions. Dent. Mater..

[14] He J., Soderling E., Osterblad M., Vallittu P.K., Lassila L.V.J. (2011). Synthesis of methacrylate monomers with antibacterial effects against S. mutans. Molecules.

[15] Zhou H., Li F., Weir M.D., Xu H.H. (2013). Dental plaque microcosm response to bonding agents containing quaternary ammonium methacrylates with different chain lengths and charge densities. J. Dent..

[16] Zhou C., Weir M.D., Zhang K., Deng D., Cheng L., Xu H.H. (2013). Synthesis of new antibacterial quaternary ammonium monomer for incorporation into CaP nanocomposite. Dent. Mater..

[17] Morones J.R., Elechiguerra J.L., Camacho A., Holt K., Kouri J.B., Ramirez J.T., Yacaman M.J. (2005). The bactericidal effect of silver nanoparticles. Nanotechnology.

[18] Slenters T.V., Hauser-Gerspach I., Daniels A.U., Fromm K.M. (2008). Silver coordination compounds as light-stable, nano-structured and anti-bacterial coatings for dental implant and restorative materials. J. Mater. Chem..

[19] Damm C., Munsted H., Rosch A. (2007). Long-term antimicrobial polyamide 6/silver nanocomposites. J. Mater. Sci..

[20] Lynch C.D., Wilson N.H. (2013). Managing the phase-down of amalgam: Part II. Implications for practising arrangements and lessons from Norway. Br. Dent. J..

[21] Cheng L., Weir M.D., Xu H.H., Antonucci J.M., Kraigsley A.M., Lin N.J., Lin-Gibson S., Zhou X. (2012). Antibacterial amorphous calcium phosphate nanocomposite with quaternary ammonium salt and silver nanoparticles. Dent. Mater..

[22] Cheng L., Zhang K., Melo M.A., Weir M.D., Zhou X., Xu H.H. (2012). Anti-biofilm dentin primer with quaternary ammonium and silver nanoparticles. J. Dent. Res..

[23] Zhang K., Melo M.A., Cheng L., Weir M.D., Bai Y., Xu H.H. (2012). Effect of quaternary ammonium and silver nanoparticle-containing adhesives on dentin bond strength and dental plaque microcosm biofilms. Dent. Mater..

[24] Ahn S.J., Lee S.J., Kook J.K., Lim B.S. (2009). Experimental antimicrobial orthodontic adhesives using nanofillers and silver nanoparticles. Dent. Mater..

[25] Akhavan A., Sodagar A., Motjahedzadeh F., Sodagar K. (2013). Investigating the effect of incorporating nanosilver/nanohydroxyapatite particles on the shear bond strength of orthodontic adhesives. Acta Odontol. Scand..

[26] Hori K., Matsumoto S. (2010). Bacterial adhesion: From mechanism to control. Biochem. Eng. J..

[27] Katsikogianni M., Missirlis Y.F. (2004). Concise review of mechanisms of bacterial adhesion to biomaterials and of techniques used in estimating bacteria-material interactions. Eur. Cells Mater..

[28] Ishihara K., Ueda T., Nakabayashi N. (1990). Preparation of phospholipid polymers and their properties as polymer hydrogel membranes. Polym. J..

[29] Ishihara K., Nomura H., Mihara T., Kurita K., Iwasaki Y., Nakabayashi N. (1998). Why do phospholipid polymers reduce protein adsorption?. J. Biomed. Mater. Res..

[30] Sibarani J., Takai M., Ishihara K. (2007). Surface modification on microfluidic devices with 2-methacryloyloxyethyl phosphorylcholine polymers for reducing unfavorable protein adsorption. Coll. Surf. B.

[31] Lewis A.L., Tolhurst L.A., Stratford P.W. (2002). Analysis of a phosphorylcholine-based polymer coating on a coronary stent pre-and post-implantation. Biomaterials.

[32] Zhang N., Chen C., Melo M.A., Bai Y., Cheng L., Xu H.H. (2015). A novel protein-repellent dental composite containing 2-methacryloyloxyethyl phosphorylcholine. Int. J. Oral Sci..

[33] Zhang N., Weir M.D., Romberg E., Bai Y., Xu H.H. (2015). Development of novel dental adhesive with double benefits of protein-repellent and antibacterial capabilities. Dent. Mater..

[34] Zhang N., Chen C., Weir M.D., Bai Y.X., Xu H.H. (2015). Antibacterial and protein-repellent orthodontic cement to combat biofilms and white spot lesions. J. Dent..

[35] Scougall-Vilchis R.J., Yamamoto S., Kitai N., Hotta M., Yamamoto K. (2007). Shear bond strength of a new fluoride-releasing orthodontic adhesive. Dent. Mater. J..

[36] Scougall-Vilchis R.J., Ohashi S., Yamamoto K. (2009). Effects of 6 self-etching primers on shear bond strength of orthodontic brackets. Am. J. Orthod. Dentofac. Orthop..

[37] Cheng H.Y., Chen C.H., Li C.L., Tsai H.H., Chou T.H., Wang W.N. (2011). Bond strength of orthodontic light-cured resin-modified glass ionomer cement. Eur. J. Orthod..

[38] Ferracane J.L., Stansbury J.W., Burke F.J. (2011). Self-adhesive resin cements—Chemistry, properties and clinical considerations. J. Oral Rehabil..

[39] Poosti M., Ramazanzadeh B., Zebarjad M., Javadzadeh P., Naderinasab M., Shakeri M.T. (2013). Shear bond strength and antibacterial effects of orthodontic composite containing TiO_2_ nanoparticles. Eur. J. Orthod..

[40] Artun J., Bergland S. (1984). Clinical trials with crystal growth conditioning as an alternative to acid-etch enamel pretreatment. Am. J. Orthod. Dentofac. Orthop..

[41] Imazato S., Tay F.R., Kaneshiro A.V., Takahashi Y., Ebisu S. (2007). An *in vivo* evaluation of bonding ability of comprehensive antibacterial adhesive system incorporating MDPB. Dent. Mater..

[42] Imazato S., Ehara A., Torii M., Ebisu S. (1998). Antibacterial activity of dentine primer containing MDPB after curing. J. Dent..

[43] Moro T., Kawaguchi H., Ishihara K., Kyomoto M., Karita T., Ito H. (2009). Wear resistance of artificial hip joints with poly(2-methacryloyloxyethyl phosphorylcholine) grafted polyethylene: Comparisons with the effect of polyethylene cross-linking and ceramic femoral heads. Biomaterials.

[44] McBain A.J. (2009). Chapter 4: *In vitro* biofilm models: An overview. Adv. Appl. Microbiol..

[45] Cheng L., Exterkate R.A., Zhou X., Li J., ten Cate J.M. (2011). Effect of Galla chinensis on growth and metabolism of microcosm biofilms. Caries Res..

[46] McBain A.J., Sissons C., Ledder R.G., Sreenivasan P.K., de Vizio W., Gilbert P. (2005). Development and characterization of a simple perfused oral microcosm. J. Appl. Microbiol..

[47] Lima J.P., Sampaio de Melo M.A., Borges F.M., Teixeira A.H., Steiner-Oliveira C., Nobre Dos Santos M., Rodrigues L.K., Zanin I.C. (2009). Evaluation of the antimicrobial effect of photodynamic antimicrobial therapy in an *in situ* model of dentine caries. Eur. J. Oral. Sci..

[48] Buyukyilmaz T., Øgaard B. (1995). Caries preventive effects of fluoride releasing materials. Adv. Dent. Res..

[49] Derks A., Katsaros C., Frencken J.E., van’t Hof M.A., Kuijpers-Jagtman A.M. (2004). Caries-inhibiting effect of preventive measures during orthodontic treatment with fixed appliances. A systematic review. Caries Res..

[50] Goda T., Konno T., Takai M., Ishihara K. (2007). Photoinduced phospholipid polymer grafting on Parylene film: Advanced lubrication and antibiofouling properties. Coll. Surf. B.

[51] Imazato S., Kinomoto Y., Tarumi H., Ebisu S., Tay F.R. (2003). Antibacterial activity and bonding characteristics of an adhesive resin containing antibacterial monomer MDPB. Dent. Mater..

[52] Beyth N., Yudovin-Farber I., Bahir R., Domb A.J., Weiss E.I. (2006). Antibacterial activity of dental composites containing quaternary ammonium polyethylenimine nanoparticles against Streptococcus mutans. Biomaterials.

[53] Murata H., Koepsel R.R., Matyjaszewski K., Russell A.J. (2007). Permanent, non-leaching antibacterial surfaces-2: How high density cationic surfaces kill bacterial cells. Biomaterials.

[54] Rai M., Yada A., Gade A. (2009). Silver nanoparticles as a new generation of antimicrobials. Biotechnol. Adv..

[55] Müller R., Eidt A., Hiller K.A., Katzur V., Subat M., Schweikl H., Imazato S., Ruhl S., Schmalz G. (2009). Influences of protein films on antibacterial or bacteria-repellent surface coatings in a model system using silicon wafers. Biomaterials.

[56] Li F., Weir M.D., Fouad A.F., Xu H.H. (2014). Effect of salivary pellicle on antibacterial activity of novel antibacterial dental adhesives using a dental plaque microcosm biofilm model. Dent. Mater..

[57] Reynolds I.R. (1975). A review of direct orthodontic bonding. Br. J. Orthod..

[58] Irie M., Suzuki K., Watts D.C. (2004). Immediate performance of self-etching *versus* system adhesives with multiple light-activated restoratives. Dent. Mater..

[59] Beyer M., Reichert J., Bossert J., Sigusch B.W., Watts D.C., Jandt K.D. (2011). Acids with an equivalent taste lead to different erosion of human dental enamel. Dent. Mater..

[60] Sharma P., Valiathan A., Arora A., Agarwal S. (2013). A comparative evaluation of the retention of metallic brackets bonded with resin-modified glass ionomer cement under different enamel preparations: A pilot study. Contemp. Clin. Dent..

